# Supplementary material for: A compelling symmetry: The extended fetuses-at-risk perspective on modal, optimal and relative birthweight and gestational age
Source: PLoS One. 2020 Nov 30;15(11):e0238673. doi: 10.1371/journal.pone.0238673 (PMC7703977; doi:10.1371/journal.pone.0238673)
Supplement: S1 Appendix — S1 Figure: Birthweight distribution in grams between 3,000 and 3,500 gms, United States, 2004–2015. S2 Figure: Contrasts of indices of interest, singletons of women 25–29 vs ≥35 years of age, United States, 2004–15. S3 Figure: Contrasts of indices of interest, singletons of White women vs Black women, United States, 2004–15. S4 Figure: Contrasts of indices of interest, singletons of low-risk women (i.e., without hypertension or diabetes) vs singletons of women with hypertension and diabetes, United States, 2004–15. S5 Figure: Contrasts of indices of interest, singletons of Black women (Panel A) and singletons of women with hypertension, United States, 2004–15. S1 Table: Numbers and rates of births and perinatal deaths among singletons of women without hypertension or diabetes, United States, 2004–2015. S2 Table: Numbers and rates of births and perinatal deaths among twins, United States, 2004–2015. S3 Table: SAS code for quantifying the first and second derivatives of the birth rate. S4 Table: Numbers of total births and perinatal deaths, and perinatal death rates in low- and high-risk cohorts, United States, 2004–2015. S5 Table: Correlation between gestational and birthweight indices, low- and high-risk cohorts, United States, 2004–2015. (PDF) [file pone.0238673.s001.pdf]

Supplementary Appendix

Table of contents

Number	Item	Page
1.	Figure legends	2
2.	S1 Figure	3
3.	S2 Figure	4
4.	S3 Figure	5
5.	S4 Figure	6
6.	S5 Figure	7
7.	S1 Table. Numbers and rates of births and perinatal deaths among singletons of women without hypertension or diabetes, United States, 2004-2015.	8
8.	S2 Table. Numbers and rates of births and perinatal deaths among twins, United States, 2004-2015.	9
9.	S3 Table. SAS code for quantifying the first and second derivatives of the birth rate.	10
10.	S4 Table. Numbers of total births and perinatal deaths, and perinatal death rates in low- and high-risk cohorts, United States, 2004-2015.	11
11.	S5 Table. Correlation between the gestational week at which the first derivative of the birth rate peaks vs the mean, mode, median and standard deviation of the birthweight distribution, optimal birthweight and the gestational week at which the first derivative of the fetuses-at-risk perinatal death rate increases in late gestation, low- and high-risk cohorts, United States, 2004-2015.	12

eFigure Legends

eFigure 1. Birthweight distribution showing numbers of births between 3,000 and 3,500 gms, United States, 2004-2015.

eFigure 2. First derivatives of the fetuses-at-risk birth and perinatal mortality rate among singletons of women 25-29 years (Panel A) and singletons of women ≥35 years of age (Panel B); gestational age distributions and births-based perinatal mortality rates among singletons of women 25-29 years (Panel C) and singletons of women ≥35 years of age (Panel D); first derivatives of the birth rate and the fetuses-at-risk perinatal mortality rate among singletons of women 25-29 vs ≥35 years (Panel E) and gestational age distributions and births-based perinatal mortality rates among singletons of women 25-29 vs ≥35 years (Panel F), United States, 2004-2015.

eFigure 3. First derivatives of the fetuses-at-risk birth and perinatal mortality rate among singletons of White women (Panel A) and singletons of Black women (Panel B); gestational age distributions and births-based perinatal mortality rates among singletons of White women (Panel C) and singletons of Black women (Panel D); first derivatives of the birth rate and the fetuses-at-risk perinatal mortality rate among singletons of White vs Black women (Panel E) and gestational age distributions and births-based perinatal mortality rates among singletons of White vs Black women (Panel F), United States, 2004-2015. Note: Although the first derivatives among Blacks were only slightly left-shifted compared with those among Whites, baseline birth rates and fetuses-at-risk perinatal mortality rates were considerably higher among Blacks (birth rates 0.68 and 1.23 at 20 and 28 weeks among Whites and 2.03 and 3.12 per 1000 fetuses-weeks at risk among Blacks, respectively; and fetuses-at-risk perinatal death rates 0.65 and 0.22 among Whites and 1.94 and 0.50 per 1000 fetus-weeks at risk among Blacks, respectively).

eFigure 4. First derivatives of the fetuses-at-risk birth and perinatal mortality rate among singletons of women without hypertension or diabetes (Panel A) and singletons of women with hypertension and diabetes (Panel B); gestational age distributions and births-based perinatal mortality rates among singletons of women without hypertension or diabetes (Panel C) and singletons of women with hypertension and diabetes (Panel D); first derivatives of the birth rate and the fetuses-at-risk perinatal mortality rate among singletons of women without hypertension or diabetes vs singletons of women with hypertension and diabetes (Panel E) and gestational age distributions and births-based perinatal mortality rates among singletons of women without hypertension or diabetes vs singletons of women with hypertension and diabetes (Panel F), United States, 2004-2015.

eFigure 5. First derivatives of the fetuses-at-risk birth and perinatal mortality rate among singletons of Black women (Panel A) and singletons of women with hypertension (Panel B); gestational age distributions and births-based perinatal mortality rates among singletons of Black women (Panel C) and singletons of women with hypertension (Panel D); first derivatives of the birth rate and the fetuses-at-risk perinatal mortality rate among singletons of Black women vs singletons of women with hypertension (Panel E) and gestational age distributions and births-based perinatal mortality rates among singletons of Black women vs singletons of women with hypertension (Panel F), United States, 2004-2015.

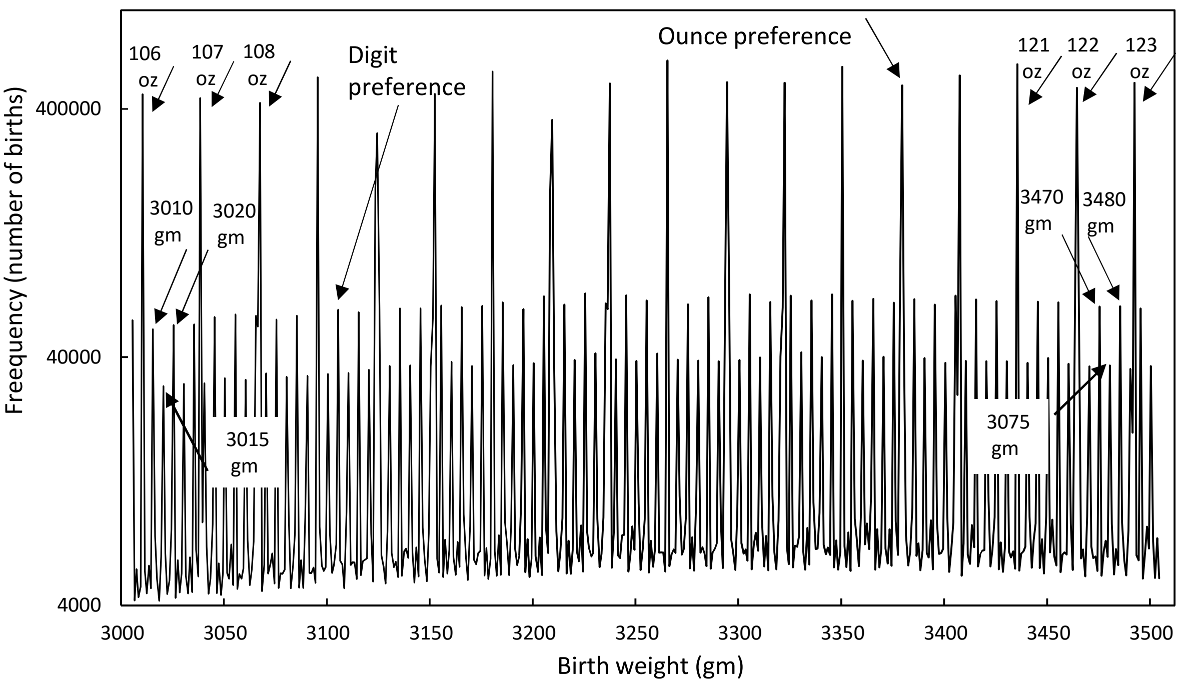
106 oz
107 oz
108 oz
Digit preference
Ounce preference
121 oz
122 oz
123 oz
3010 gm
3020 gm
3470 gm
3480 gm
3015 gm
3075 gm
400000
40000
4000
Freequency (number of births)
3000
3050
3100
3150
3200
3250
3300
3350
3400
3450
3500
Birth weight (gm)

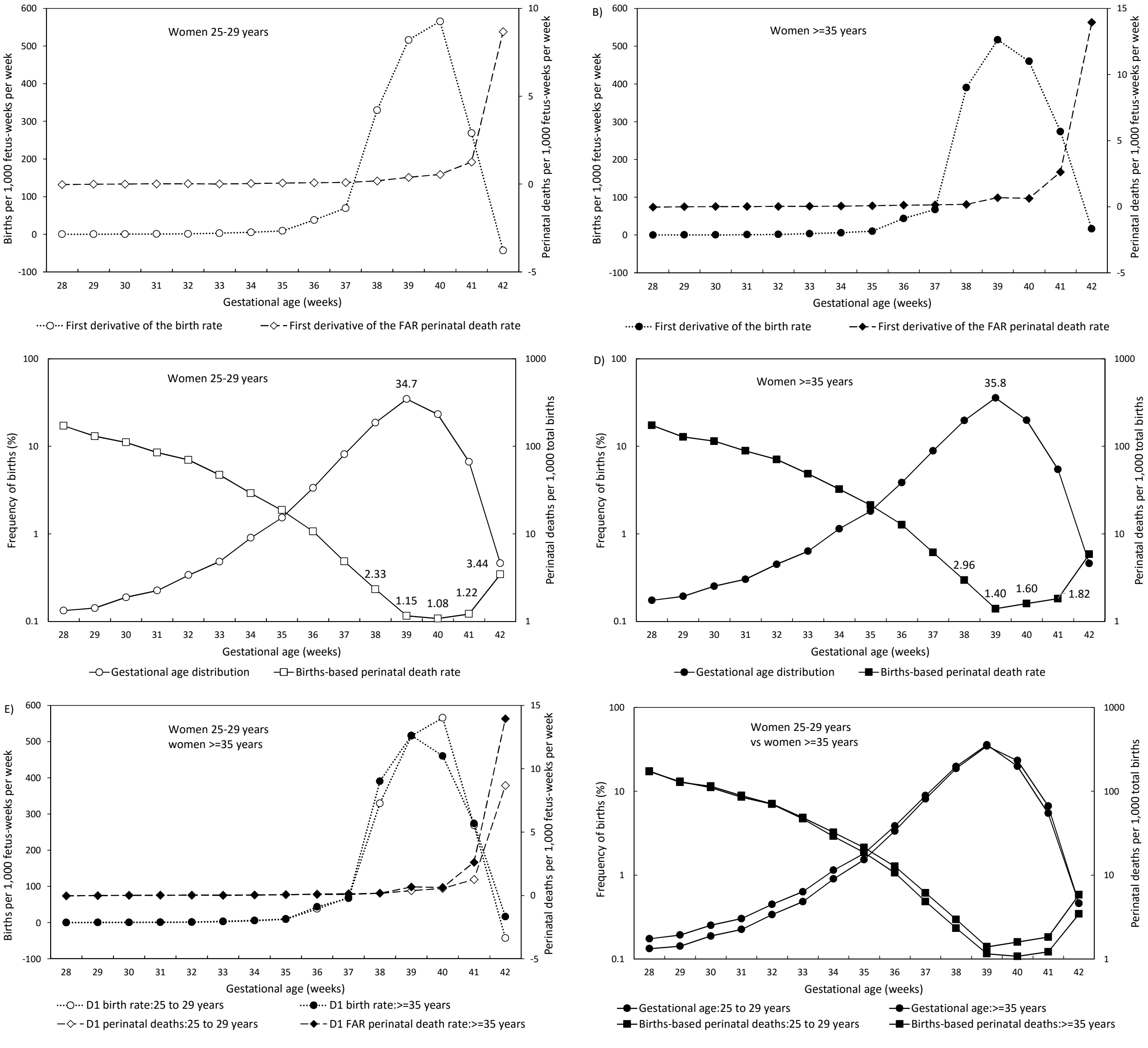
B)
D)
E)
Women 25-29 years
Women >=35 years
Women 25-29 years
women >=35 years
Women 25-29 years
vs women >=35 years
Births per 1,000 fetus-weeks per week
Perinatal deaths per 1,000 fetus-weeks per week
Frequency of births (%)
Perinatal deaths per 1,000 total births
Gestational age (weeks)
First derivative of the birth rate
First derivative of the FAR perinatal death rate
Gestational age distribution
Births-based perinatal death rate
34.7
2.33
1.15
1.08
1.22
3.44
35.8
2.96
1.40
1.60
1.82
D1 birth rate:25 to 29 years
D1 birth rate:>=35 years
D1 perinatal deaths:25 to 29 years
D1 FAR perinatal death rate:>=35 years
Gestational age:25 to 29 years
Gestational age:>=35 years
Births-based perinatal deaths:25 to 29 years
Births-based perinatal deaths:>=35 years

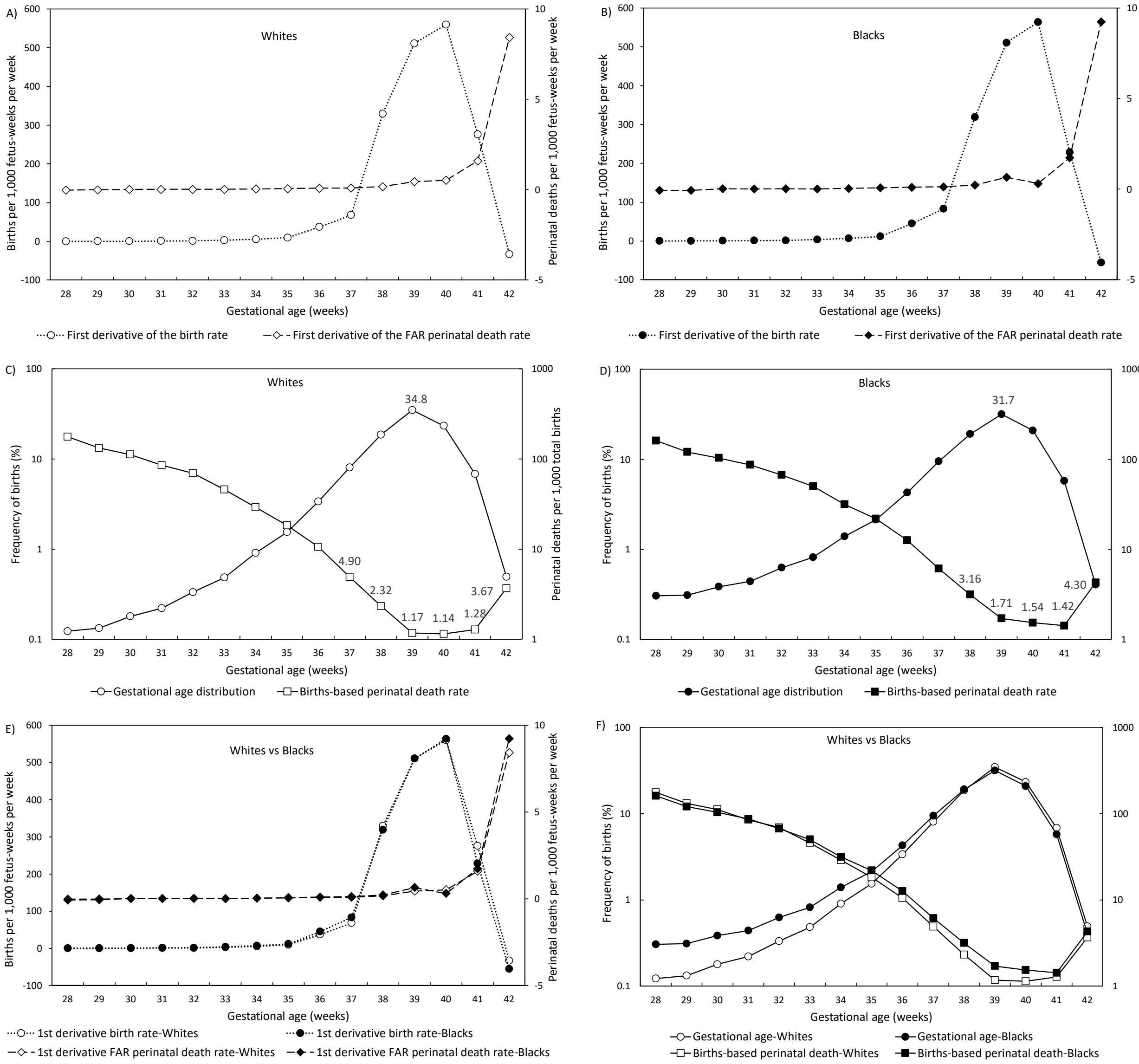
A)
Whites
Births per 1,000 fetus-weeks per week
Perinatal deaths per 1,000 fetus-weeks per week
Gestational age (weeks)
First derivative of the birth rate
First derivative of the FAR perinatal death rate
B)
Blacks
Births per 1,000 fetus-weeks per week
Perinatal deaths per 1,000 fetus-weeks per week
Gestational age (weeks)
First derivative of the birth rate
First derivative of the FAR perinatal death rate
C)
Whites
34.8
4.90
2.32
1.17
1.14
1.28
3.67
Frequency of births (%)
Perinatal deaths per 1,000 total births
Gestational age (weeks)
Gestational age distribution
Births-based perinatal death rate
D)
Blacks
31.7
3.16
1.71
1.54
1.42
4.30
Frequency of births (%)
Perinatal deaths per 1,000 total births
Gestational age (weeks)
Gestational age distribution
Births-based perinatal death rate
E)
Whites vs Blacks
Births per 1,000 fetus-weeks per week
Perinatal deaths per 1,000 fetus-weeks per week
Gestational age (weeks)
1st derivative birth rate-Whites
1st derivative birth rate-Blacks
1st derivative FAR perinatal death rate-Whites
1st derivative FAR perinatal death rate-Blacks
F)
Whites vs Blacks
Frequency of births (%)
Perinatal deaths per 1,000 total births
Gestational age (weeks)
Gestational age-Whites
Gestational age-Blacks
Births-based perinatal death-Whites
Births-based perinatal death-Blacks

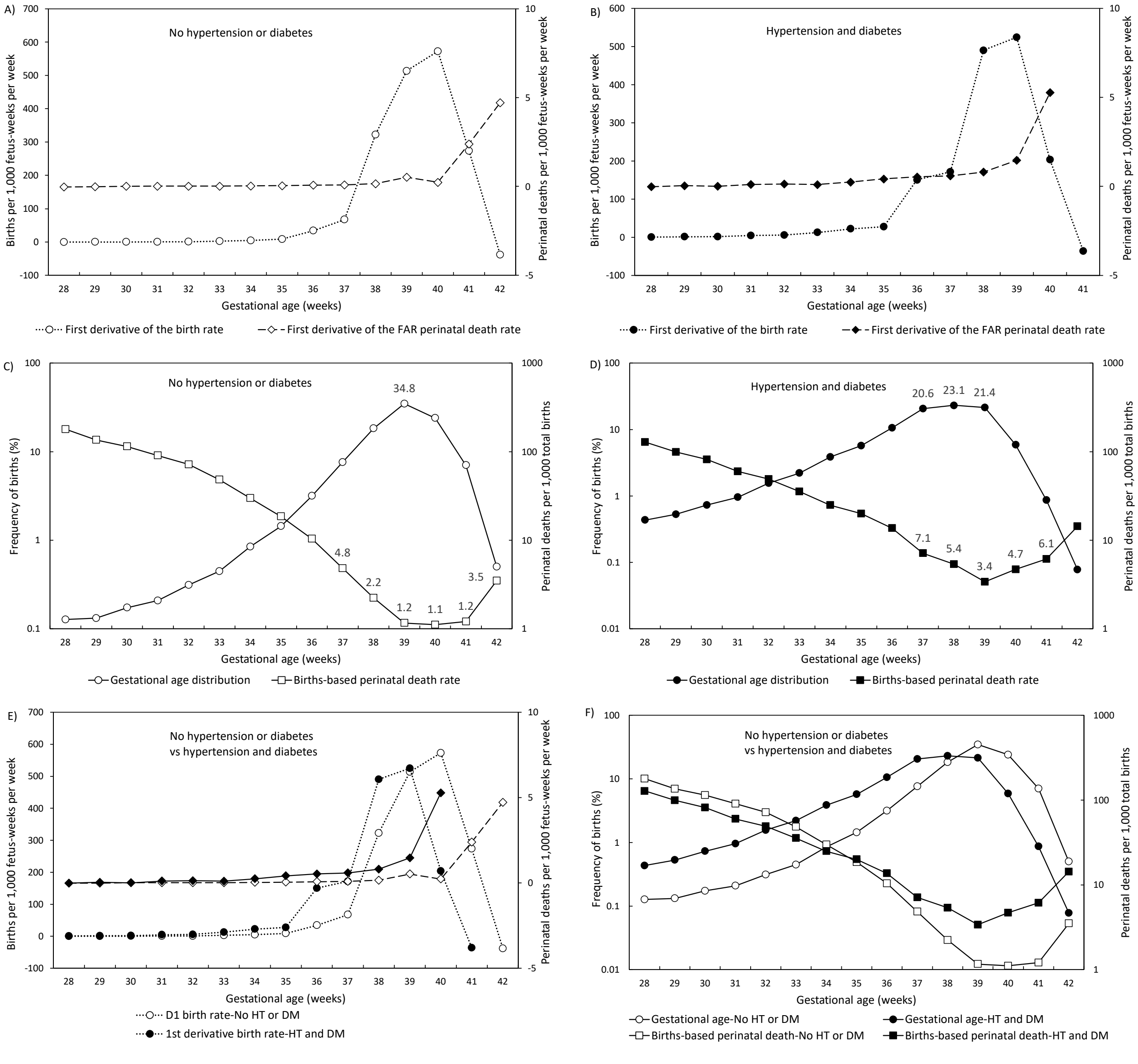
A)
No hypertension or diabetes
Births per 1,000 fetus-weeks per week
Perinatal deaths per 1,000 fetus-weeks per week
Gestational age (weeks)
First derivative of the birth rate
First derivative of the FAR perinatal death rate
B)
Hypertension and diabetes
Births per 1,000 fetus-weeks per week
Perinatal deaths per 1,000 fetus-weeks per week
Gestational age (weeks)
First derivative of the birth rate
First derivative of the FAR perinatal death rate
C)
No hypertension or diabetes
34.8
4.8
2.2
1.2
1.1
1.2
3.5
Frequency of births (%)
Perinatal deaths per 1,000 total births
Gestational age (weeks)
Gestational age distribution
Births-based perinatal death rate
D)
Hypertension and diabetes
20.6
23.1
21.4
7.1
5.4
3.4
4.7
6.1
Frequency of births (%)
Perinatal deaths per 1,000 total births
Gestational age (weeks)
Gestational age distribution
Births-based perinatal death rate
E)
No hypertension or diabetes
vs hypertension and diabetes
Births per 1,000 fetus-weeks per week
Perinatal deaths per 1,000 fetus-weeks per week
Gestational age (weeks)
D1 birth rate-No HT or DM
1st derivative birth rate-HT and DM
F)
No hypertension or diabetes
vs hypertension and diabetes
Frequency of births (%)
Perinatal deaths per 1,000 total births
Gestational age (weeks)
Gestational age-No HT or DM
Gestational age-HT and DM
Births-based perinatal death-No HT or DM
Births-based perinatal death-HT and DM

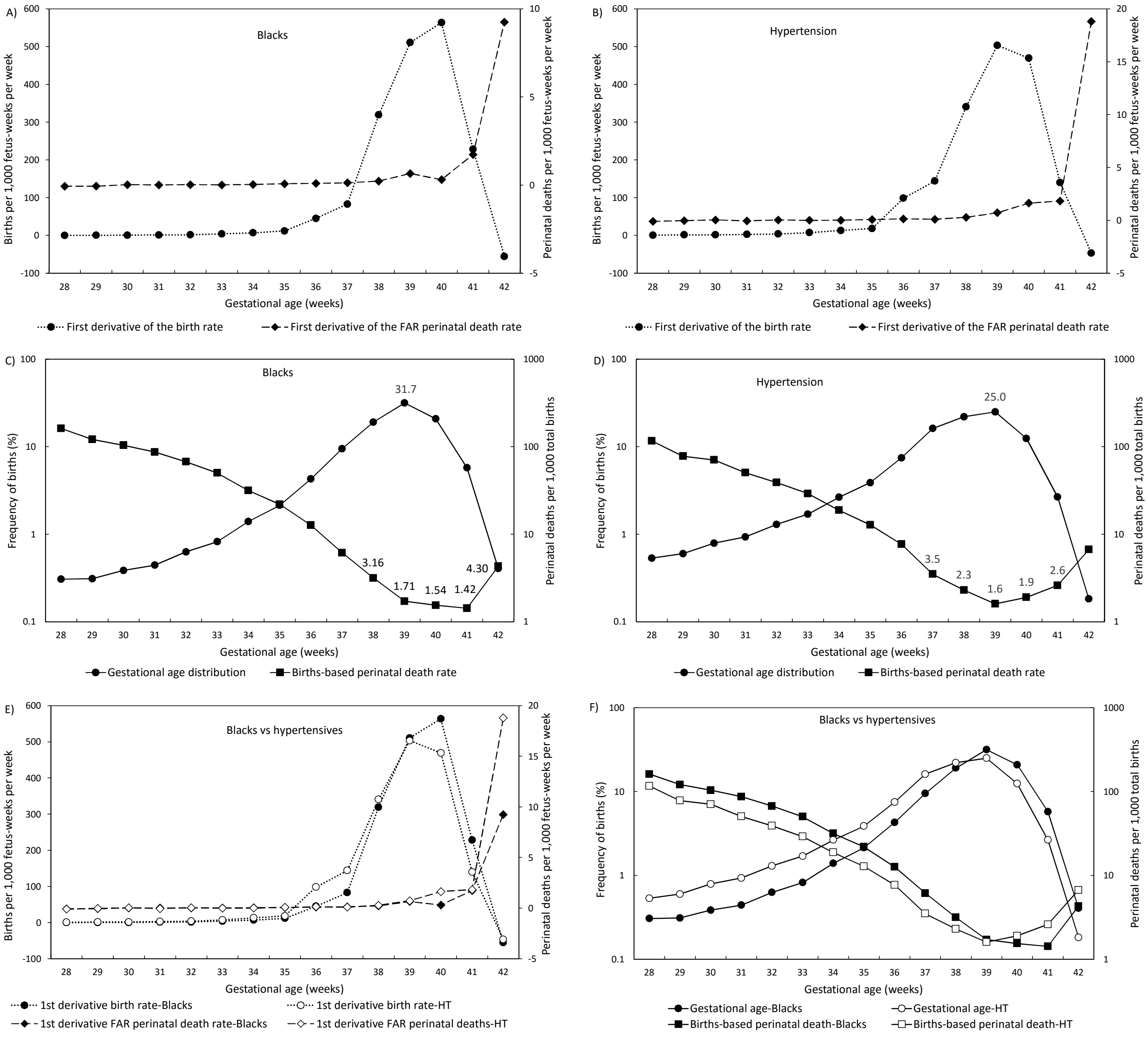
A)
Blacks
Births per 1,000 fetus-weeks per week
Perinatal deaths per 1,000 fetus-weeks per week
Gestational age (weeks)
First derivative of the birth rate
First derivative of the FAR perinatal death rate
B)
Hypertension
Births per 1,000 fetus-weeks per week
Perinatal deaths per 1,000 fetus-weeks per week
Gestational age (weeks)
First derivative of the birth rate
First derivative of the FAR perinatal death rate
C)
Blacks
31.7
3.16
1.71
1.54
1.42
4.30
Frequency of births (%)
Perinatal deaths per 1,000 total births
Gestational age (weeks)
Gestational age distribution
Births-based perinatal death rate
D)
Hypertension
25.0
3.5
2.3
1.6
1.9
2.6
Frequency of births (%)
Perinatal deaths per 1,000 total births
Gestational age (weeks)
Gestational age distribution
Births-based perinatal death rate
E)
Blacks vs hypertensives
Births per 1,000 fetus-weeks per week
Perinatal deaths per 1,000 fetus-weeks per week
Gestational age (weeks)
1st derivative birth rate-Blacks
1st derivative birth rate-HT
1st derivative FAR perinatal death rate-Blacks
1st derivative FAR perinatal deaths-HT
F)
Blacks vs hypertensives
Frequency of births (%)
Perinatal deaths per 1,000 total births
Gestational age (weeks)
Gestational age-Blacks
Gestational age-HT
Births-based perinatal death-Blacks
Births-based perinatal death-HT

S1 Table. Numbers and rates of births and perinatal deaths among singletons of women without hypertension or diabetes, United States, 2004-2015.

Gestational age (weeks)	Total births	Perinatal deaths	Fetuses at risk	Fetus weeks at risk	Birth rate per 1,000 fetuses at risk	Birth rate per 1,000 fetus-weeks at risk	Births-based PMR per 1,000 total births	Fetuses at risk PMR per 1,000 fetus-weeks at risk
a	b	c	d	e	f=b/d	g=b/e	h=(c*1000/b)	i=(c*1000/e)
20	36,180	34,300	41,201,466	41,183,376	0.88	0.88	948.0	0.83
21	37,520	35,772	41,165,286	41,146,526	0.91	0.91	953.4	0.87
22	39,463	36,533	41,127,766	41,108,035	0.9	0.96	925.8	0.89
23	38,265	27,731	41,088,303	41,069,171	0.93	0.93	724.7	0.68
24	42,724	19,567	41,050,038	41,028,676	1.04	1.04	458.0	0.48
25	41,457	13,038	41,007,314	40,986,586	1.01	1.01	314.5	0.32
26	43,124	10,863	40,965,857	40,944,295	1.05	1.05	251.9	0.27
27	44,944	9,087	40,922,733	40,900,261	1.10	1.10	202.2	0.22
28	52,454	9,390	40,877,789	40,851,562	1.28	1.28	179.0	0.23
29	54,409	7,404	40,825,335	40,798,131	1.33	1.33	136.1	0.18
30	71,468	8,190	40,770,926	40,735,192	1.75	1.75	114.6	0.20
31	86,129	7,811	40,699,458	40,656,394	2.12	2.12	90.7	0.19
32	129,004	9,273	40,613,329	40,548,827	3.18	3.18	71.9	0.23
33	184,318	8,914	40,484,325	40,392,166	4.55	4.56	48.4	0.22
34	349,561	10,458	40,300,007	40,125,227	8.67	8.71	29.9	0.26
35	594,459	11,034	39,950,446	39,653,217	14.9	15.0	18.6	0.28
36	1,306,146	13,584	39,355,987	38,702,914	33.2	33.8	10.4	0.35
37	3,141,439	15,183	38,049,841	36,479,122	82.6	86.1	4.8	0.42
38	7,555,000	16,905	34,908,402	31,130,902	216.4	242.7	2.2	0.54
39	14,334,53	16,670	27,353,402	20,186,326	524.0	710.1	1.2	0.83
40	9,897,310	11,002	13,019,249	8,070,594	760.2	1,226.3	1.1	1.36
41	2,901,886	3,514	3,121,939	1,670,996	929.5	1,736.6	1.2	2.10
42	206,584	724	220,053	116,761	938.8	1,769.3	3.5	6.20
43	13,469	126	13,469	6,735	1000.0	2,000.0	9.4	18.71
Total	41,201,466	337,073	-	-	-	-	8.2	-

PMR denotes perinatal mortality rate. Fetuses at risk at any gestational week include all total births at that week and all subsequent gestational weeks. Fetus-weeks at risk represent an average of the fetuses at that gestational week and the subsequent week.

S2 Table. Numbers and rates of births and perinatal deaths among twins, United States, 2004-2015.

Gestational age (weeks)	Total births	Perinatal deaths	Fetuses at risk	Fetus weeks at risk	Birth rate per 1,000 fetuses at risk	Birth rate per 1,000 fetus-weeks at risk	Births-based PMR per 1,000 total births	Fetuses at risk PMR per 1,000 fetus-weeks at risk
a	b	c	d	E	f=b/d	g=b/e	h=(c*1000/b)	i=(c*1000/e)
20	6,826	6,520	1,586,008	1,582,595	4.30	4.31	955.2	4.12
21	7,583	7,224	1,579,182	1,575,391	4.80	4.81	952.7	4.59
22	8,638	7,943	1,571,599	1,567,280	5.50	5.51	919.5	5.07
23	9,336	6,405	1,562,961	1,558,293	5.97	5.99	686.1	4.11
24	10,871	4,638	1,553,625	1,548,190	7.00	7.02	426.6	3.00
25	11,101	2,866	1,542,754	1,537,204	7.20	7.22	258.2	1.86
26	12,895	2,331	1,531,653	1,525,206	8.42	8.45	180.8	1.53
27	15,067	1,695	1,518,758	1,511,225	9.92	9.97	112.5	1.12
28	18,668	1,617	1,503,691	1,494,357	12.4	12.5	86.6	1.08
29	21,256	1,202	1,485,023	1,474,395	14.3	14.4	56.5	0.82
30	29,054	1,073	1,463,767	1,449,240	19.9	20.1	36.9	0.74
31	39,351	1,012	1,434,713	1,415,038	27.4	27.8	25.7	0.72
32	62,710	1,180	1,395,362	1,364,007	44.9	46.0	18.8	0.87
33	86,437	1,057	1,332,652	1,289,434	64.9	67.0	12.2	0.82
34	144,566	1,283	1,246,215	1,173,932	116.0	123.2	8.9	1.09
35	199,090	1,089	1,101,649	1,002,104	180.7	19867	5.5	1.09
36	283,562	1,131	902,559	760,778	314.2	372.7	4.0	1.49
37	324,277	1,078	618,997	456,859	523.9	709.8	3.3	2.36
38	231,515	607	294,720	178,963	785.5	1,293.7	2.6	3.39
39	48,276	207	63,205	39,067	763.8	1,235.7	4.3	5.30
40	13,401	93	14,929	8,229	897.7	1,628.6	6.9	11.3
41	1,251	19	1,528	903	818.7	1,386.2	15.2	21.1
42	220	6	277	167	794.2	1,317.4	27.3	35.9
43	57	1	57	29	1,000.0	2,000.0	17.5	35.1
Total	1,586,008	52,277	-	-	-	-	33.0	-

PMR denotes perinatal mortality rate. Fetuses at risk at any gestational week include all total births at that week and all subsequent gestational weeks. Fetus-weeks at risk represent an average of the fetuses at that gestational week and the subsequent week.

S3 Table. SAS code for quantifying the first and second derivatives of the birth rate.

```
PROC IMPORT OUT= WORK.abc
            DATAFILE= "C:\Users\Pap2\Births"
            DBMS=XLSX;
RUN;
**** Note: The file ‘Births’ contains birth rates (Number of births per 1000
fetus weeks) by gestational week. Variable names for gestational age in weeks
and birth rate are Gest and BRD, respectively;
proc gplot data=abc;
  title 'Original Series';
  plot BRD*Gest;
  run;
proc sort data=abc;
  by Gest;
  run;
**** Compute the first derivative of the fitted spline;
proc expand data=abc outest=two out=three;
convert BRD=slope/observed=(beginning,derivative);
id Gest;
  run;

**** Clip the slope when abs(slope)>20 so that the graph appears clearly;
data four;
  set three;
  deriv1=slope;
  if slope >100000 then deriv1=100000;
  if slope <-100000 then deriv1=-100000;
  run;
**** Create the graph of Y vs X and DERIV1 vs X;
proc gplot data=four;
  title 'Series and First Derivative';
  title2 'DERIV1 is clipped at -100000/100000';
  plot (BRD deriv1)*GEST/overlay;
  run;
**** Compute the second derivative;
proc expand data=four out=five;
  convert slope=deriv/observed=(beginning,derivative);  id GEST;
  run;
**** Clip the values of the second derivative;
data six;
  set five;
  deriv2=deriv;
  if deriv >150000 then deriv2=150000;
  if deriv <-150000 then deriv2=-150000;
  run;
proc gplot data=six(firstobs=2);
  title 'Series, First Derivative and Second Derivative';
  title2 'DERIV1 is clipped at -100000/100000';
  title3 'DERIV2 is clipped at -150000/150000';
  plot (BRD deriv1 deriv2)*GEST / overlay;
  proc print; var GEST BRD deriv1 deriv2;
  run;
```

S4 Table. Numbers of total births and perinatal deaths, and perinatal death rates in low- and high-risk cohorts, United States, 2004-2015.

Cohort	Number of total births	Number of perinatal deaths	Perinatal death rate per 1,000 total births
Singletons of women without HT and DM*	41,201,466	337,073	8.2
Singletons of women with HT	2,281,595	30,039	13.2
Singletons of women with DM	1,954,975	17,531	9.0
Singletons of women with HT and DM	357,188	6,207	17.4
Twins	1,586,008	52,277	33.0
Triplets	63,521	4,599	72.4
Singletons of White women	35,065,915	262,314	7.5
Singletons of Black women	7,335,068	119,859	16.3
Singletons of women aged 25-29 years	12,997,353	104,057	8.0
Singletons of women aged ≥35 years	6,593,277	70,289	10.7
Singletons of women with previous preterm birth	597,329	5,308†	8.9†
Singletons of women without a previous preterm birth	26,434,667	78,669†	3.0†

*HT denotes hypertension and DM denotes diabetes mellitus.

†Restricted to neonatal deaths (information on previous preterm birth not available in fetal death files).

S5 Table. Correlation between the gestational week at which the first derivative of the birth rate peaks vs the mean, mode, median and standard deviation of the birthweight distribution, optimal birthweight and the gestational week at which the first derivative of the fetuses-at-risk perinatal death rate increases sharply, low- and high-risk cohorts, United States, 2004-2015.

Cohort	Peak in the 1st derivative of the birth rate (weeks)	Birthweight distribution						Late gestation increase in 1st derivative of the FAR perinatal death rate
		Mean	SD	Mode		Median	Optimal	
				Observed	Modeled			
Singletons of women – No HT or DM	40	3,312	545	3,260	3,326	3,335	3,892	40
Singletons of HT women	39	3,040	729	3,260	3,185	3,140	3,949	41
Singletons of DM women	39	3,385	596	3,430	3,354	3,395	3,807	41
Singletons of HT and DM women	39	3,182	766	3,260	3,269	3,238	3,864	39
Twins	37	2,334	630	2,466	2,562	2,415	2,902	38
Triplets	35	1,654	569	1,899	1,854	1,701	1,968	35
Younger mother (25-29 years)	40	3,321	552	3,260	3,326	3,345	3,864	41
Older mothers (≥35 years)	39	3,336	594	3,430	3,354	3,374	3,949	40
Whites	40	3,344	547	3,430	3,354	3,373	3,864	40
Blacks	40	3,117	616	3,260	3,213	3,175	3,751	40
Previous preterm birth	39	3,007	708	3,090	3,185	3,090	3,581	41
No previous preterm birth	40	3,308	554	3,260	3,326	3,330	3,864	40
Pearson r (with D1 peak of birth rate)[a]	1.00	0.95	-0.08	0.93	0.95	0.95	0.94	0.86
95% confidence interval	-	0.82, 0.99	-0.63, 0.52	0.75, 0.98	0.83, 0.99	0.82, 0.99	0.79, 0.98	0.58, 0.96
P value	-	<0.001	0.80	<0.001	<0.001	<0.001	<0.001	<0.001
Pearson r (GA at D1 rise in late gestation)[b]							0.90	1.00
95% confidence interval							0.69, 0.97	-
P value							<0.001	-

D1 denotes the first derivative; SD standard deviation; FAR fetuses at risk; HT hypertension; and DM diabetes mellitus.
Optimal birthweight refers to the point in the birthweight distribution at which the perinatal death rate is lowest (see text).

[a] Pearson correlation between the gestational week at which the first derivative (D1) of the birth rate peaks and other indices (n=12).

[b] Pearson correlation between the gestational week at which the first derivative (D1) of the fetuses-at-risk perinatal death rate increases sharply and optimal birthweight (n=12).
