# Supplementary material for: A compelling symmetry: The extended fetuses-at-risk perspective on modal, optimal and relative birthweight and gestational age
Source: PLoS One. 2020 Nov 30;15(11):e0238673. doi: 10.1371/journal.pone.0238673 (PMC7703977; doi:10.1371/journal.pone.0238673)
Supplement: Attachment — Submitted filename: Response to Reviewers.docx [file pone.0238673.s002.docx]

**Response to Reviewers’ comments**

**Comments to the Author**
1. Is the manuscript technically sound, and do the data support the conclusions?
The manuscript must describe a technically sound piece of scientific research with data that supports the conclusions. Experiments must have been conducted rigorously, with appropriate controls, replication, and sample sizes. The conclusions must be drawn appropriately based on the data presented.

Reviewer #1: Yes

Reviewer #2: Yes

 2. Has the statistical analysis been performed appropriately and rigorously?

 Reviewer #1: Yes

Reviewer #2: Yes

 3. Have the authors made all data underlying the findings in their manuscript fully available?
The [PLOS Data policy](http://www.plosone.org/static/policies.action#sharing) requires authors to make all data underlying the findings described in their manuscript fully available without restriction, with rare exception (please refer to the Data Availability Statement in the manuscript PDF file). The data should be provided as part of the manuscript or its supporting information, or deposited to a public repository. For example, in addition to summary statistics, the data points behind means, medians and variance measures should be available. If there are restrictions on publicly sharing data—e.g. participant privacy or use of data from a third party—those must be specified.

Reviewer #1: Yes

Reviewer #2: Yes

4. Is the manuscript presented in an intelligible fashion and written in standard English?
PLOS ONE does not copyedit accepted manuscripts, so the language in submitted articles must be clear, correct, and unambiguous. Any typographical or grammatical errors should be corrected at revision, so please note any specific errors here.

Reviewer #1: Yes

Reviewer #2: Yes

5. Review Comments to the Author
Please use the space provided to explain your answers to the questions above. You may also include additional comments for the author, including concerns about dual publication, research ethics, or publication ethics. (Please upload your review as an attachment if it exceeds 20,000 characters).

**Reviewer #1:**

**Comment:** The research article is informative but diagrams would look better if multiple colors could be used.

**Response:** The Figures have been revised and colors have been used to distinguish the curves in the graphs.

**Comment:** Please explain why acceleration in the birth rate peaks earlier in higher-risk cohorts compared with lower-risk cohorts.

**Response:** The revised manuscript now includes an explanation for why the birth rate peaks earlier in higher risk cohorts (Page 13-14 lines 285-91)

“It has been suggested that the greater acceleration in the birth rate of higher-risk cohorts represents an exaggerated, hypersensitivity‐type response to adverse influences in pregnancy, and could reflect an evolutionary mechanism that prioritises maternal survival in the face of potential threats to fetal well‐being [29]. However, the mechanism underlying peak acceleration in the birth rate, and its subsequent decline is unclear and one postulated explanation involves a depletion of susceptibles: pregnancies that reach late gestation are less responsive to hormonal and other triggers that initiate parturition [29].”

**Reviewer #2:**

**Comment:** This manuscript presents further analysis on a larger data set from NCHS birth-death linkage records with the purpose of presenting an analysis of perinatal deaths taking into account the change of such deaths by week of gestation.
The authors obtained an estimate on change of rates from cubic spline or polynomial curve fitting of the rates using the number of fetuses at risk, and describes an assessment of perinatal deaths taking into account differences in birth rates by gestational age. The approach proposed by the authors is similar to the Bongaarts and Feeney approach to demographic phenomena and it was received with skepticism and not widely adopted to the assessment of cross-sectional data such as those presented here. The CDC NVSS calls the data birth cohort, but they are in nature cross-sectional data, as opposed to true birth cohort studies that collect information as early as possible during pregnancy. The authors may want to address this limitation of their data. An example of a birth cohort study can be found in the reference below.
1. Olsen J, Melbye M, Olsen SF, Sørensen TI, Aaby P, Andersen AM, Taxbøl D, Hansen KD, Juhl M, Schow TB, Sørensen HT, Andresen J, Mortensen EL, Olesen AW, Søndergaard C. The Danish National Birth Cohort--its background, structure and aim. Scand J Public Health. 2001 Dec;29(4):300-7. doi: 10.1177/14034948010290040201. PMID: 11775787.

### Response: The data used for the study was obtained from the period linked birth-infant death data files of the CDC’s National Center for Health Statistics (available at https://www.cdc.gov /nchs/data_access/vitalstatsonline.htm). The period linked birth-infant death files are different from the NVSS birth files (also available in the same data repository). The distinction between these files arises because the period linked birth-infant death files contain linked information from the birth file and the (fetal and infant) death files. For instance, the NVSS birth file for 2017, includes information on all live births that occurred in the United States in 2017. On the other hand, the 2017 period linked birth-infant death file includes information from the

### a) NVSS births file with information on all live births that occurred in 2017

### b) fetal death file which includes information on all stillbirths that occurred in 2017

### c) death file with information on all infants deaths that occurred in 2017

### The 3 files are linked with the intent of documenting the longitudinal experience of all viable births in 2017, with the events of interest restricted to fetal deaths that occur after 20 weeks’ gestation, and infant deaths i.e., deaths that occur within 1 year after birth. Since all live births and stillbirths that occur in 2017 are included in the file, this file does constitute the full 2017 birth cohort denominator. The longitudinal follow up for infant deaths that occur in 2018 to live births occurring in 2017 is not included; this is approximated by including infant deaths in 2017 among live births that occurred in 2016. Note that the cohort birth-infant death files for 2017 include the longitudinal follow up into 2018 and hence availability of these files is delayed by 1 year. In short, the period linked birth-infant death files document the longitudinal experience of a birth cohort’s experience with regard to perinatal mortality and infant mortality.

### The period and cohort effects to which the Reviewer alludes are well understood within the perinatal epidemiology community and are generally referred to under the concept termed ‘age-period-cohort’ effects. This paper deals primarily with (gestational) age-specific effects among different groups/cohort and period and cohort effects are not explored. Period effects and cohort effects would have shaped some of the phenomena explored in the study: increases in iatrogenic early delivery have increased late preterm birth in recent decades, and obstetric and neonatal care has substantially reduced perinatal death rates. Cohort effects in terms of alterations to maternal health have probably been less obvious, though general improvements in maternal health in recent cohorts and increases in the fertility of women with chronic disease have likely played a role in shaping perinatal mortality trends. However, these phenomena are not likely to have a material impact in shaping the relative (gestational) age-specific patterns of birth and perinatal death rates among contrasted groups e.g., low-risk singletons vs singletons of mothers with hypertension since all contrasts of intersecting mortality curves were within a given period and cohort. The revised manuscript include a sentence regarding this issue in the limitations section of the manuscript (Page 16, line 334-37).

### “Thus, changes in obstetric and neonatal care, which have impacted birth and perinatal mortality rates over recent decades, likely did not compromise the relative gestational age-specific analyses in this study as contrasted cohorts (e.g., singletons of low-risk women vs twins) would have been affected almost uniformly by period and cohort effects.”

6. PLOS authors have the option to publish the peer review history of their article ([what does this mean?](https://journals.plos.org/plosone/s/editorial-and-peer-review-process#loc-peer-review-history)). If published, this will include your full peer review and any attached files.
If you choose “no”, your identity will remain anonymous but your review may still be made public.
**Do you want your identity to be public for this peer review?** For information about this choice, including consent withdrawal, please see our [Privacy Policy](https://www.plos.org/privacy-policy).

Reviewer #1: **Yes:** Farzana Ahmed

Reviewer #2: **Yes:** Victor M Cardenas

[NOTE: If reviewer comments were submitted as an attachment file, they will be attached to this email and accessible via the submission site. Please log into your account, locate the manuscript record, and check for the action link "View Attachments". If this link does not appear, there are no attachment files.]

While revising your submission, please upload your figure files to the Preflight Analysis and Conversion Engine (PACE) digital diagnostic tool, <https://pacev2.apexcovantage.com/>. PACE helps ensure that figures meet PLOS requirements. To use PACE, you must first register as a user. Registration is free. Then, login and navigate to the UPLOAD tab, where you will find detailed instructions on how to use the tool. If you encounter any issues or have any questions when using PACE, please email PLOS at [figures@plos.org](mailto:figures@plos.org). Please note that Supporting Information files do not need this step.

**Response:** This has been done.
