# Supplementary material for: A compelling symmetry: The extended fetuses-at-risk perspective on modal, optimal and relative birthweight and gestational age
Source: PLoS One. 2020 Nov 30;15(11):e0238673. doi: 10.1371/journal.pone.0238673 (PMC7703977; doi:10.1371/journal.pone.0238673)
Supplement: Attachment — Submitted filename: Response to ReviewersNov2020.docx [file pone.0238673.s003.docx]

**Response to Reviewers’ comments**

**Comments to the Author**
1. If the authors have adequately addressed your comments raised in a previous round of review and you feel that this manuscript is now acceptable for publication, you may indicate that here to bypass the “Comments to the Author” section, enter your conflict of interest statement in the “Confidential to Editor” section, and submit your "Accept" recommendation.

Reviewer #1: All comments have been addressed

Reviewer #2: (No Response)

**Response:** No response required.

 2. Is the manuscript technically sound, and do the data support the conclusions?
The manuscript must describe a technically sound piece of scientific research with data that supports the conclusions. Experiments must have been conducted rigorously, with appropriate controls, replication, and sample sizes. The conclusions must be drawn appropriately based on the data presented.

Reviewer #1: Yes

Reviewer #2: (No Response)

**Response:** No response required.

3. Has the statistical analysis been performed appropriately and rigorously?

 Reviewer #1: Yes

Reviewer #2: (No Response)

**Response:** No response required.

 4. Have the authors made all data underlying the findings in their manuscript fully available?
The [PLOS Data policy](http://www.plosone.org/static/policies.action#sharing) requires authors to make all data underlying the findings described in their manuscript fully available without restriction, with rare exception (please refer to the Data Availability Statement in the manuscript PDF file). The data should be provided as part of the manuscript or its supporting information, or deposited to a public repository. For example, in addition to summary statistics, the data points behind means, medians and variance measures should be available. If there are restrictions on publicly sharing data—e.g. participant privacy or use of data from a third party—those must be specified.

Reviewer #1: Yes

Reviewer #2: (No Response)

**Response:** No response required.

5. Is the manuscript presented in an intelligible fashion and written in standard English?
PLOS ONE does not copyedit accepted manuscripts, so the language in submitted articles must be clear, correct, and unambiguous. Any typographical or grammatical errors should be corrected at revision, so please note any specific errors here.

Reviewer #1: Yes

Reviewer #2: (No Response)

**Response:** No response required.

6. Review Comments to the Author
Please use the space provided to explain your answers to the questions above. You may also include additional comments for the author, including concerns about dual publication, research ethics, or publication ethics. (Please upload your review as an attachment if it exceeds 20,000 characters)

Reviewer #1: (No Response)

Reviewer #2: The author should acknowledge the limitation of the data to deaths occurring <20 weeks of gestation (about 20% of pregnancies end up in pregnancy losses, the births may not truly represent the denominator (persons at-risk). The data is birth-death linkage cross-sectional in nature not from a cohort study.

**Response:** A sentence has been added to the Limitations section of the manuscript which acknowledges that the study population was restricted to births at and above 20 weeks’ gestation and that pregnancy losses occurring before 20 weeks were not included in the study’s fetuses-at-risk denominators. Additionally, the revised manuscript includes a sentence stating that the period linked births-infant deaths files are essentially cross sectional in nature (unlike the cohort linked births-infant death files).

Page 15, line 322-325

“The study population was restricted to births 20-43 week’s gestation and pregnancy losses that occurred prior to 20 weeks were not included in the study’s fetuses-at-risk denominators. Further, the period linked births-infant deaths files used for the study are essentially cross-sectional in nature (unlike the cohort linked births-infant death files).”
